# The Important Role of Interleukin-2 in COVID-19

**DOI:** 10.1155/2023/7097329

**Published:** 2023-08-22

**Authors:** Leila Ghanbari Naeini, Laleh Abbasi, Farzaneh Karimi, Pajman Kokabian, Fahimeh Abdi Abyaneh, Delaram Naderi

**Affiliations:** ^1^Gulf Medical University, Ajman, UAE; ^2^Guilan University of Medical Sciences, Rasht, Iran; ^3^Behbahan Faculty of Medical Sciences, Behbahan, Iran; ^4^School of Medicine, Shahid Beheshti University of Medical Sciences, Tehran, Iran; ^5^Kashan University of Medical Sciences, Kashan, Iran; ^6^Faculty of Medicine, Iran University of Medical Sciences (IUMS), Tehran, Iran

## Abstract

There is controversial literature about the effects of the interleukin-2 (IL-2) cytokine family in COVID-19 pathogenesis and immunity. So we aimed to identify the potential in the role of the IL-2 family in COVID-19. A narrative review search was done through online databases, including PubMed, Scopus, and Web of Science. The search deadline was up to December 2022. We applied no time limits for the searching strategy. After retrieving articles from the databases, the authors summarized the data into two data extraction tables. The first data extraction table described the changes in the IL-2 cytokine family in COVID-19 and the second table described the therapeutic interventions targeting IL-2 family cytokines. The results of the literature on the role of the IL-2 cytokine family do not show a singular rule. IL-2 cytokine family can change during severe acute respiratory syndrome coronavirus 2 (SARS-CoV-2) infection. Some studies suggest that IL-2 cytokine family rise during the infection and cause severe inflammatory response and cytokine storm. These cytokines are shown to be increased in immunocompromised patients and worsen their prognosis. In individuals without underlying disease, the upregulation of the IL-2 family shows the clinical outcome of the disease and rises with disease severity. However, some other studies show that these cytokines do not significantly change. IL-2 cytokine family is mostly upregulated in healthy individuals who had vaccination, but immunocompromised patients did not show significant changes after a single dose of vaccines, which shows that these patients need booster doses for efficient immunity. IL-2 cytokine family can also be used as immunotherapy agents in COVID-19.

## 1. Introduction

An unusual case of pneumonia was reported in China toward the end of 2019, and tests confirmed it to be a new coronavirus disease named coronavirus disease 2019 (COVID-19) [1]. COVID-19, caused by the severe acute respiratory syndrome coronavirus 2 (SARS-CoV-2) [2], has become a global pandemic burden with devastating health, social, and economic repercussions [2, 3]. Since then, researchers have struggled to determine the possible molecular mechanisms which interfere with disease pathogenicity to find the pharmacologic targets [4–6]. The term “cytokine storm” refers to the excessive release of cytokines caused by the activation of immune system cells in response to a viral invasion [7, 8]. Cytokine storm might have a role in poor outcomes in patients with COVID-19, and immune responses were associated with the emergence and progression of the virus [9]. Interleukins (ILs) were shown to have a crucial role in the development of COVID-19, as demonstrated by the growing body of research on this topic. Serum interleukin levels were significantly higher in severely ill COVID-19 patients than in those with milder illnesses [10].

Moreover, there were variations in cytokine levels among COVID-19 individuals who did and did not survive [11]. IL-2 is a cytokine that is responsible for the regulation of white blood cells, most often lymphocytes. IL-2 binds to IL-2 receptors, expressed on lymphocytes, to exert its actions. Activated CD4+ T cells and CD8+ T cells are the primary producers of IL-2 [12]. IL-2 shares a common structure with other cytokines, including IL-4, 7, 9, 15, and 21. They all possess a four-alpha helix bundle [12]. Previous research on the role of IL-2 in viral respiratory infection shows that the IL-2 levels can be used to determine the prognosis of lung damage in influenza A patients. Considering the evidence on the effects of IL-2 in viral infections, this study aims to review the molecular mechanisms and immunomodulatory effects of IL-2 in COVID-19 and its targeting agents [13].

A narrative review search was done through online databases, including PubMed, Scopus, and Web of Science. The following keywords were used to do the searching strategy: (IL-2 OR IL-2R OR IL-4 OR IL-7 OR IL-9 OR IL-15 OR IL-21) AND (COVID-19 OR SARS-CoV-2). The inclusion criteria were the original studies on the role of the IL-2 cytokine family in COVID-19. The exclusion criteria were non-English studies, studies without full text, letters, and conference papers. We applied no time limits for the searching strategy. The search deadline was up to December 2022.

## 2. Immunologic Background of COVID-19

The COVID-19 pandemic has created an unprecedented global health crisis, with millions of people infected and hundreds of thousands of deaths worldwide [14]. COVID-19 is caused by a novel coronavirus called SARS-CoV-2 [15], which primarily affects the respiratory system and can cause a wide range of clinical manifestations, from asymptomatic infection to severe respiratory illness and death [4, 16]. The immune system plays a crucial role in the pathogenesis of COVID-19, both in terms of the host response to the virus and the development of disease [3, 17]. The innate immune system is the first line of defense against invading pathogens, including SARS-CoV-2. The innate immune response is initiated by the recognition of pathogen-associated molecular patterns (PAMPs) by pattern recognition receptors (PRRs) expressed on the surface of immune cells [18]. These PRRs can recognize viral RNA [19], lipopolysaccharides [20], and other pathogen-associated molecules and initiate signaling cascades that lead to the production of pro-inflammatory cytokines, chemokines, and other immune mediators. In COVID-19, the innate immune response is activated early after infection and plays a critical role in controlling viral replication and spread [21]. However, the sustained and excessive production of pro-inflammatory cytokines, including interleukin-6 (IL-6), tumor necrosis factor-alpha (TNF-*α*), and interferon-gamma (IFN-*γ*), can also lead to severe tissue damage and organ dysfunction, known as a cytokine storm. In COVID-19, the adaptive immune response is critical for the clearance of the virus and the prevention of reinfection [22, 23]. The presence of neutralizing antibodies and virus-specific T cells is associated with a better prognosis and the development of protective immunity. However, the dysregulation of the adaptive immune response can also contribute to the pathogenesis of severe disease [24]. Cytokines are produced by various immune cells, including T cells, B cells, macrophages, and dendritic cells, and they act on a range of target cells, including immune cells, endothelial cells, and epithelial cells [25]. In COVID-19 patients, IL-1 family cytokines are elevated, and they are thought to contribute to the cytokine storm observed in severe cases of COVID-19. IL-1 inhibitors, such as anakinra, have been used in different investigations to reduce the inflammatory response and improve outcomes in COVID-19 patients [26]. IL-6 is another cytokine that is elevated in COVID-19 patients and is thought to play a critical role in the cytokine storm observed in the severe cases of COVID-19. IL-6 is produced by immune cells, including T cells and macrophages, and it promotes inflammation and immune activation. IL-6 inhibitors, such as tocilizumab and sarilumab, have been used in clinical trials to reduce inflammation and improve outcomes in COVID-19 patients [27]. IL-10 is an anti-inflammatory cytokine that can regulate the immune response and prevent tissue damage. In COVID-19 patients, IL-10 levels are reduced, leading to dysregulated immune responses and tissue damage. IL-10 has been proposed as a potential therapeutic target for COVID-19, and IL-10 administration has been shown to reduce the inflammation and improve outcomes in animal models of COVID-19 [28].

**Figure 1:**
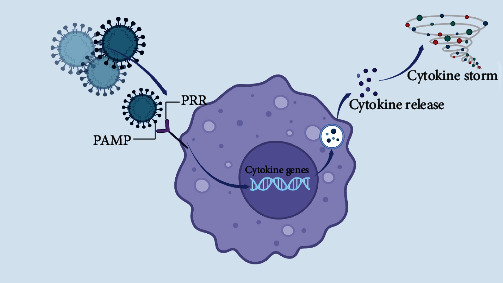
The general mechanism underlying immune system response to SARS-CoV-2 invasion. When the PAMP of the virus attaches to the PRR, the pattern of the virus is detected by immune system. This leads to a signal transduction to the genome of immune cells and as a result, it can activate the cytokine genes. Activation of cytokine genes lead to cytokine release. If the cytokine release happens excessively, the cytokine storm happens.

Overall, interleukins play a critical role in the immune response to viral infections, including COVID-19. Dysregulation of interleukin production can lead to a cytokine storm and tissue damage, contributing to the severity of COVID-19. Interleukin inhibitors have been used in clinical trials to reduce the inflammation and improve outcomes in the COVID-19 patients, and they represent a promising therapeutic approach for COVID-19. However, further research is needed to fully understand the role of interleukins in COVID-19 pathogenesis and to develop the effective therapeutic strategies (Figure 1).

## 3. IL-2 Family Overview

IL-2 is a cytokine within the IL-2 cytokine family in which each member contains a four alpha-helix bundle. Other members of this family include IL-4, IL-7, IL-9, IL-15, and IL-21. IL-2 signals via the IL-2 receptor, a complex comprised of three chains named alpha (CD25), beta (CD122), and gamma (CD132). All family members participate in the gamma chain [12]. The IL-2 receptor (IL-2R) subunit binds IL-2 with modest affinity. Due to its short intracellular chain, the interaction between IL-2 and CD25 alone does not result in signal transduction, but it increases IL-2R affinity by hundredfold [29]. T cell signaling depends on the heterodimerization of the subunits of IL-2R. IL-2 may initiate signaling via the intermediate-affinity dimeric CD122/CD132 IL-2R or the high-affinity trimeric CD25/CD122/CD132 IL-2R. Dimeric IL-2R is expressed by memory CD8+ T cells and natural killer (NK) cells, whereas regulatory T cells and activated T cells exhibit large quantities of trimeric IL-2R [30].

T helper 2 (Th2) cells are derived from naïve helper T cells (Th0 cells), and IL-4 is a cytokine that induces this development [31, 32]. Th2 cells undergo a positive feedback loop wherein they create more IL-4 after being activated by the cytokine. Th2 cells, mast cells, basophils, and eosinophils are the primary cells responsible for IL-4 production [33]. One-half of the structure of IL-4 is dominated by a four alpha-helix bundle with a left-handed twist; the helices are antiparallel with two overhand connections, and they fall into a two-stranded antiparallel beta-sheet. IL-4 has a tight, globular fold, stabilized by three disulfide bonds [34].

IL-7 is a hematopoietic growth factor released by bone marrow and thymus stromal cells. It is also generated by keratinocytes, dendritic cells, hepatocytes, neurons, and epithelial cells, but not by normal lymphocytes [35]. The 3D structure of IL-7 was determined by X-ray. The IL-7 receptor is a heterodimer composed of the alpha and gamma subunits of the IL-7 receptor [36]. IL-7 is a cytokine that promotes the growth of B and T cells. IL-7 and the hepatocyte growth factor (HGF) create a heterodimer that acts as a growth factor for pre–pro-B cells. During early T cell development, this cytokine is discovered to be a cofactor for V(D)J rearrangement of the T cell receptor beta (TCR-*ß*) [37].

IL-9 is generated at varying levels, including NKT cells, mast cells, Th2, Treg, Th17, ILC2, and Th9 cells. Th9 cells are the most critical CD4+ T cells that generate IL-9 [38]. In order to transmit signals, the gamma chain of the IL-2 receptor interacts with the IL-9 receptor, which is a member of the hemopoietin receptor superfamily. Although the Jak-STAT pathway has been the focus of much signal transduction research due to its centrality in several IL-9 bioactivities, the 4PS/IRS2 adaptor protein may also play a crucial role in IL-9 signaling [39]. IL-15 is another cytokine from the IL-2 cytokine family. A combination comprising the IL-2/IL-15 receptor beta chain (CD122) and the typical gamma chain mediates the binding and signaling of IL-15, as it does for IL-2 (gamma-C, CD132) [40]. When confronted with a viral infection, mononuclear phagocytes (and other cells) release IL-15 (es). Naturally occurring killer cells (NKCs), whose primary function is to eliminate virus-infected cells, are stimulated to multiply in response to this cytokine [41].

IL-21 is a cytokine that stimulates the cell division and proliferation in its target cells. IL-21 has significant regulatory effects on immune cells, such as NK and cytotoxic T cells, which may eliminate virally infected or malignant cells [42, 43]. T cells, B cells, and NK cells all have IL-21 receptors (IL-21R) on their surfaces. Similar to cytokine receptors like IL-2R [44], the IL-21 receptor (IL-21R) dimerizes with the common gamma chain (c) to bind IL-21 [45, 46].

Inflammatory signaling pathways can affect the expression of IL-2 cytokines by activating transcription factors that bind to the IL-2 gene promoter and enhance transcription. One such pathway is the NF-*κ*B pathway, which is activated by various stimuli, including pro-inflammatory cytokines, such as IL-1, TNF-*α*, and IL-6 [47]. Activation of the NF-*κ*B pathway leads to the phosphorylation and degradation of its inhibitor, I*κ*B, and the translocation of NF-*κ*B to the nucleus, where it binds to the promoter region of the IL-2 gene and enhances its transcription [48, 49]. Additionally, other signaling pathways, such as MAPK and PI3K, can also affect the expression of IL-2 cytokines. Activation of these pathways leads to the activation of transcription factors, such as AP-1 and NFAT, which can also bind to the promoter region of the IL-2 gene and enhance its transcription [49].

## 4. A Brief Description of the Role of the IL-2 Family in Viral Infection

Cytokines play a significant role in viral infection by regulating cell signaling pathways. It has been shown that cytokines can be involved directly in disease pathogenesis [50]. Viruses led to the induction of cytokine genes, which can help viral genome replication. Some cytokines are known as viral inhibitors, especially IL-12 and interferons, which downregulate viral replication. However, it has been demonstrated that cytokines from the IL-2 family, such as IL-4, can increase the pathogenicity of the virus [51]. Research has indicated the remarkable role of IL-2 on HIV-infected T cells. This cytokine was demonstrated to increase the viral replication and its receptors, including CCR5 and CXCR4. Additionally, patients with HIV were shown to have IL-2 and decreased expression of transcription factors which repress viral replication inhibitors [52]. It has been demonstrated that IL-7 could exhibit antiviral properties by targeting CD8+ cells and affecting their function. IL-7 was used as an antiviral treatment in mice infected with viruses. The results showed that IL-7 can lead to a significant proliferation of B and T cells and played a role in viral clearance in mice after the cytokine therapy. It was also shown that IL-7 could upregulate T-cell populations and increase their transportation from the thymus [53]. The role of IL-9 in viral infections was not clearly defined by the previous literature. However, it was shown that IL-9 could modulate CD4+ responses in some viral infections, including respiratory syncytial virus infection [54] and coxsackievirus B3-induced myocarditis [55]. IL-15 has been known for its regulatory role in antiviral responses of NK cells and CD8+ cells in activated immune reactions. The expression of IL-15 is affected by infectious agents.

Then IL-15 can play its regulatory role through modulation of these cells in the infection site, where NK cells and T-cells migrate and expand. IL-15 upregulation also increases the phagocytosis of viral pathogens by inducing proinflammatory factor release and chemotaxis of monocytes and macrophages [41]. IL-21 is a crucial element in the management of chronic viral infections. Mice lacking IL-21 (or IL-21R) that had been chronically infected with LCMV (lymphocytic choriomeningitis virus) were less successful in fighting off the illness.

Furthermore, LCMV-specific CD8+ T cells were depleted to a greater extent in mice with impaired IL-21 signaling, indicating that IL-21 produced by CD4+ T cells is necessary for sustained CD8+ T-cell effector activity and, consequently, for maintaining immunity to resolve persistent viral infection [56]. It has been shown that HIV-specific CD4+ T cells from “HIV controllers” (rare individuals who do not progress to AIDS by controlling the virus replication without treatment) are able to produce significantly more IL-21 than those of progressors. Furthermore, IL-21-generating virus-specific CD8 T cells were also seen in HIV-infected individuals [57].

## 5. The Role of the IL-2 Family in COVID-19

A deep review of literature in the online databases showed that the exact mechanism of action of the IL-2 cytokine family in the pathogenesis of COVID-19 was not clearly defined. However, there are some evidences which could demonstrate the changes in cytokines with or without an intervention in COVID-19 patients.

Some studies investigated cytokine changes in patients with COVID-19 who suffered from underlying diseases. Maranatha et al. [58] designed a study to identify how TNF-*α*, TGF- *β*1, amphiregulin, IL-2, and estimated glomerular filtration rate changed in COVID-19 patients with pulmonary fibrosis. They found that none of the cytokines except TNF-*α* was associated with COVID-19 severity and pulmonary fibrosis. This study demonstrated that IL-2 did not correlate with COVID-19 severity and pulmonary fibrosis. A similar study by Ghazavi et al. [59] also showed that there was no change in IL-9, a cytokine from IL-2 family, in COVID-19 patients. They showed that only TGF-*β* can act as a prognostic factor for COVID-19. However other studies showed that TNF-*α* changes during inflammation can destroy the epithelial tissues [60].

On the other hand, Tappe et al. [61] showed that T-cell immunity (IFN-*γ*, IL-2, CXCL9, and CXCL10 cytokines) and innate immunity were diminished in patients with COVID-19. As a result, this downregulation could increase the risk of superinfection in these patients by weakening the antifungal immune mechanisms [61]. While Tappe et al's. [61] study shows a downregulation in IL-2 cytokine, other studies represent a different result in other patients with different underlying diseases. In a study by Rodríguez-Morales et al. [62], in two patients with a brain tumor who had COVID-19, the cytokine profile was upregulated. Increased amounts of IL-13, IFN-*γ*, and IL-2 in these patients disrupted the blood-brain barrier by activating the microglia and an exacerbated immune response.

Other studies investigated the changes in cytokines in patients with COVID-19 without any underlying diseases. In these studies, critical COVID-19 outcomes [63], extended hospitalization and lymphopenia in COVID-19 patients [64], and poor prognosis and disease severity [65] were associated with a significant increase in IL-2 cytokine family profile, including IL-2, IL-4, and IL-15. Perpiñan et al. [66] showed that resistin and IL-15 are predictors of invasive mechanical ventilation in COVID-19 patients. They also mentioned that cytokine storm is a predictor of severity in viral pneumonia in patients with metabolic syndrome. However, there was no association between increased levels of IL-15 and metabolic syndrome. In a similar study, Vaz de Paula et al. [67] showed that cytokine storms caused by SARS-CoV-2 could enhance the alveolar damage and increase the cell proliferation of fibrosis tissue in the lungs. IL-4 was significantly increased in lung autopsies. IL-4 can increase lung damage by activating Th1, Th2, and Th17 responses, and it is the key cytokine in inflammatory response [68].

**Figure 2:**
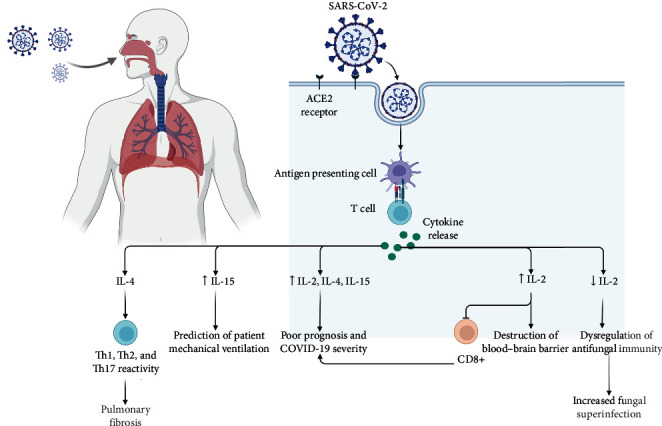
IL-2 cytokine family changes after SARS-CoV-2 infection.

Previously, the effect of IL-2 on cellular immunity was investigated. It was noted that IL-2/2R could activate JAK1–STAT5 signaling and therefore activate the CD8+ cells [69]. In a study by Shi et al. [70], it was shown that IL-2 was significantly upregulated in the plasma of COVID-19 patients. However, the expression of IL-2R and JAK1–STAT5 pathway was decreased in COVID-19 patients with severe clinical manifestations. This group demonstrated that the inhibition of IL-2R and an increase in IL-2 levels could lead to a downregulation of CD8+ cells. As a result, it can be noted that targeting IL-2 could effectively prevent fatal outcomes of the disease and increase the quality of life of patients with COVID-19 (Table 1, Figure 2).

## 6. IL-2 Family in COVID-19 Infection

There is limited research on the role of IL-2 in different COVID-19 variants. However, some studies have suggested that IL-2 levels may differ in different COVID-19 variants. In Sadhu et al. [71] study, it was showed that IL-9 aggravates SARS-CoV-2 infection. They found that IL-9 level is associated with moderate disease severity [72].

Several preventive and therapeutic strategies have been found for COVID-19 [3]. Targeting interleukins involved in COVID-19 has been a therapeutic option for COVID-19 since the start of the outbreak. Inhibition of these molecules could, most of the time, lead to the prevention of fatal disease [73, 74].

### 6.1. IL-2 Family in COVID-19 Infection: The Role of Nutritional Supplements

Nutrition supplements have always been a choice for the prevention of diseases. In COVID-19 era, the use of these supplements have increased and the need to investigate the effectiveness of these supplements became vital [75]. Nutrition Bio-shield Superfood (NBS) is a wheat germ-based product that contains alpha-linolenic acid, glutathione, fibers, vitamins, minerals, and phytochemicals. It was previously shown that this dietary supplement could help to increase white blood cells and neutrophil count, which can protect the body against pathogenic agents [76]. Jalilian et al. [77] demonstrated that NBS (2 g/day for 4 week) could effectively decrease the cytokines profile of the patients, including IL-2, IL-6, and TNF-*α* was decreased significantly. So the inflammatory process of the disease was controlled to some extent. Although this supplement was an effective anti-inflammatory agent, it could not change the overall disease severity. This indicates the adjuvant role of supplements, but not their therapeutic activity alone. Palmitoylethanolamide (PEA) is a novel supplementary, endocannabinoid-like lipid mediator with a background of neuroprotective, immunoprotective, and anti-inflammatory activity [78]. Levagen+ is a supplementary drug based on PEA. In a randomized trial performed by Fessler et al. [79], the effect of Levagen (600 mg twice daily for 4 weeks) was investigated on COVID-19 patients and compared to the control group. The participants' inflammatory cytokine expression (sP-selectin, IL-1*β*, and IL-2) was evaluated. The results showed that this supplement could significantly prevent the inflammatory process in nonhospitalized patients.

### 6.2. Targeting IL-2 Family in COVID-19 Infection: The Role of Vaccination

Vaccination is now available for the prevention of COVID-19 around the world. mRNA, viral vector, and inactivated vaccines are examples of the technologies used to develop an efficient vaccine against COVID-19. However, the immunity caused by these vaccines is still questionable [3]. The effects of COVID-19 on the expression of IL-2 family cytokines were evaluated in several studies after different vaccine doses.

While the elevated levels of IL-2 family in COVID-19 is pathogenic and might cause severe inflammation in patients, the elevated IL-2 in individuals who are not currently infected with the virus can boost their cellular immunity by the activation of T cells by IL-2 family. The aim of the clinical researchers on the effects of COVID-19 vaccination is to increase cellular immunity to win the battle against the virus. Third/fourth vaccination with Comirnaty® and Spikevax [80], two doses of COVID-19 mRNA-based vaccines (BNT162b2 (Pfizer-BioNTech) or mRNA-1273 (Moderna)) [81], single dose COVID-19 vaccination with inactivated vaccines (CoronaVac, BBIBP-CorV, or WIBP-CorV) [82], and first and second dose of BNT162b2 mRNA [83] showed a significant in IL-2 family expression, including IL-2 and IL-15 which lead to partial viral immunity in vaccinated individuals. However, in some studies, no changes were seen in the IL-2 family profile, and as a result, boosted immunity after single dose COVID-19 vaccination with BNT162b2 in patients with a rheumatic disease [84]. So the efficacy of a single dose of the COVID-19 vaccine might not be satisfying, and the administration of a booster might help these patients have better immunity against SARS-CoV-2 [85].

### 6.3. Targeting IL-2 Family in COVID-19 Infection: The Role of Immunotherapy

Targeting severe inflammatory responses to a viral infection is an option to prevent the destructive effects of cytokine storm, disease morbidity, and mortality in patients with COVID-19 [86]. The innate immunity cells, including NK cells, are not specified to identify SARS-CoV-2. Neutralizing antibodies can bind to the subunits of the entering receptor of SARS-CoV-2, named angiotensin-converting enzyme 2 (ACE2). Although the NK cells cannot express ACE2 specified receptors on their cellular surface, finding a way to engineer these cells to express ACE2 might be an excellent option to prevent infection [87]. In a study by Lu et al. [88], NK cells were isolated and chimeric antigen receptor (CAR) NK cells were engineered, and a soluble human IL-15 was added to this complex. Administration of this complex to the A549 cell line and Humanized K18-hACE2 mouse model resulted in enhanced NK cell viral protection and increased expression of inflammatory cytokines including TNF-*α* and IFN-*γ*. The mentioned experiment can be used in further clinical investigations in patients with COVID-19 who face severe and drug-resistant disease. Another immunotherapy for COVID-19 with a member of the IL-2 cytokine family, IL-7, was a trial performed by Laterre et al. [89]. IL-7 is a cytokine which plays a role in increasing lymphocytes. It is currently available immunotherapy previously used in oncology. IL-7 therapy can also lead to decreased viral load and prognosis in fatal viral infections [90]. So, in immunosuppressed COVID-19 patients, immunotherapy with IL-7 can restore immunity and result in better clinical outcomes [89] (Table 2).

## 7. Deep Insight in the Molecular Mechanisms of IL-2 Family

**Figure 3:**
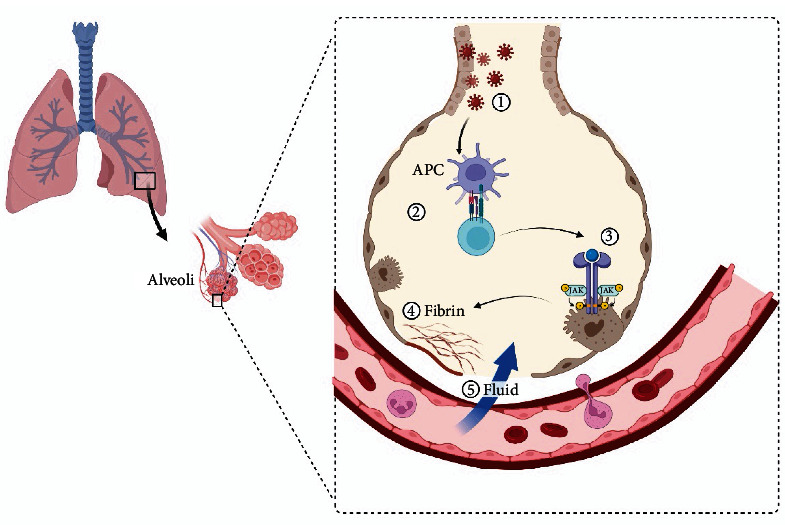
1: Coronavirus infects lung cells; 2: antigen presenting cells (APCs) attach to T helper and T cytotoxic cells, which in turn produce IL-2 cytokine family cytokines, creating a cycle of inflammation that damages the lung cells; 3–5: lung fibrosis can occur through the formation of fibrin. Blood vessels are filled with fluid leading to respiratory failure and patient poor prognosis.

The Janus Kinase (JAK)/Signal Transducers and Activators of Transcription (STAT) is a signaling pathway enrolled in the SARS-CoV-2 cytokine storm and inflammatory reactions. JAK/STAT pathway is regulated through a cytokine receptor signaling. In cells with no inflammatory activity, STAT proteins with SH2 domain are not active. After the initiation of inflammatory response to a stimulant, STAT proteins bind to phosphortyrosine of the receptor through JAK signaling. The phosphorylated STAT proteins leads to dimerization of STAT. This dimerization helps the STAT proteins to translocate into the nucleus and, as a result, immune-related changes, including apoptosis and cell differentiation occur [2, 91]. When SARS-CoV-2 enters the lung through inspiration of viral particles, the antigen presenting cells (APCs) present the antigen to t helper and cytotoxic cells. Then these cells produce the IL-2 cytokine family. When IL-2 cytokine family, including IL-2, IL-4, IL7, IL-9, IL-15, and IL-21 attach to the IL-2R, the JAK/STAT will be activated. The activation of JAK/STAT pathway leads to an inflammatory response including fibrin formation and lung fibrosis, activation of a cytokine cascade, and fluid leak from the vessels into the alveoli, which in turn causes acute respiratory distress (Figure 3) [92].

## 8. The Role of IL-2 in the Diagnosis and Prognosis of COVID-19

Biomarkers are the molecules which are used in diagnosis and prognosis of a disease. There are several factors for diagnosis and prognosis of the COVID-19 [17, 93]. The immunologic response of the patients with COVID-19 was investigated in several literature to show the impact of IL-2 family for predicting the prognosis of the disease. To achieve this goal, the serum levels of these cytokines were compared in patients with severe COVID-19 and nonsevere COVID-19. The results of the studies showed a controversial concept which requires more attention for further research. In a study on 69 patients with prolonged COVID-19 showed that, high IL-2R in serum is correlated with a prolonged disease in COVID-19 patients. The results of this study indicate that neutrophils and T cells are enrolled in the evolution of COVID-19 [94]. On the counter, in a study on 115 patients, the blood sample tests demonstrated that serum IL-2 was lower in patients with severe or moderate COVID-19 than individuals with asymptomatic and mild COVID-19 [95]. A study by Hou et al. [96] also evaluated biomarkers for the early detection of severe COVID-19. On the counter, this study showed that IL-2R is a significant cytokine which needs critical attention for the detection of severe COVID-19 cases and could predict the clinical progress of the patients. An analysis of cytokines Iraqi patients with COVID-19 demonstrated that the serum levels of IL-4 were significantly higher in patients with severe form of COVID-19 [97]. Serum IL-7, a member of IL-2 family was also compared in patients with both severe and nonsevere COVID-19. The results of the study showed that IL-7 high levels is associated with a more severe disease [98].

A systematic review and meta-analysis showed that serum level of IL-2 is indicative of COVID-19 infection compared to the healthy individuals. However, the results of that study showed that IL-2 and IL-4 are not significantly altered in severe and nonsevere COVID-19 patients [99]. Another study evaluated cytokine profile of patients with the severe and nonsevere COVID-19. The results also showed that the level of IL-9 did not differ among controls and COVID-19 patients [59].

COVID-19 has been associated with an increased risk of venous thromboembolism (VTE), which is thought to be related to a complex interplay between inflammatory cytokines, coagulation factors, and endothelial dysfunction. IL-2 may contribute to this process by promoting the activation of both immune cells and platelets, thereby promoting inflammation and thrombosis [100]. It has been demonstrated that some pro-inflammatory cytokines could potentially induce coagulation and lead to thrombosis. IL-2 can activate cytokines which are enrolled in the impairment of anticoagulation system in the intima of the vessels which leads to the induction of coagulation system [101].

## 9. Conclusions and Perspectives

The results of the literature on the role of the IL-2 cytokine family do not show a singular rule. IL-2 cytokine family can change during SARS-CoV-2 infection under different circumstances. So investigating the association between the expression of IL-2 in different clinical situations is vital.

Some studies suggest that the IL-2 cytokine family rises during the infection and cause severe inflammatory response and cytokine storm. Cytokine storm is a common cause of death in patients with COVID-19 [102]. Many studies suggest that the prevention of cytokine storm can be performed through inhibition of many different cytokines, including IL-6 [103], TNF-*α* [104], and IL-1 family [11]. These cytokines are shown to be increased in immunocompromised patients and worsen their prognosis. IL-2 family is responsible for severe inflammatory responses in COVID-19, which can similarly affect the prognosis of patients with underlying diseases, including patients with fungal infections, cancer, and metabolic disorders.

In individuals without underlying disease, the upregulation of the IL-2 family shows the clinical outcome of the disease and rises with disease severity. However, some other studies show that these cytokines do not significantly change. IL-2 cytokine family is predominantly upregulated in healthy individuals who had the vaccination, but immunocompromised patients did not show significant changes after a single dose of vaccines, which shows that these patients need booster doses for efficient immunity. IL-2 cytokine family can also be used as immunotherapy agents to prevent the severe outcomes of COVID-19.

The future perspectives on the role of the IL-2 cytokine family in COVID-19 are promising but require further investigation. First, there is a need for more extensive research to investigate the association between the expression of IL-2 in different clinical situations, including patients with underlying diseases, those with severe or mild symptoms, and vaccinated individuals. This will help researchers to identify the specific patient populations that are at-risk of developing severe inflammatory responses and cytokine storms, and develop targeted therapies to prevent such outcomes.

Second, cytokine storm is a common cause of death in COVID-19 patients, and many studies suggest that the prevention of cytokine storm can be performed through inhibition of various cytokines, including the IL-2 family. Therefore, future research should focus on identifying specific inhibitors, including monoclonal antibodies and corticosteroids that can effectively control the upregulation of the IL-2 cytokine family and prevent severe inflammatory responses in COVID-19 patients [105, 106].

Third, the upregulation of the IL-2 cytokine family has been shown to be a clinical outcome of the disease and is associated with disease severity. Therefore, IL-2 cytokine family can be used as a potential biomarker to monitor the disease progression and treatment efficacy.

Finally, IL-2 cytokine family can be used as mmunotherapy agents to prevent the severe outcomes of COVID-19. Future research should focus on developing the IL-2-based immunotherapies that can effectively modulate the immune response and prevent severe inflammatory responses in COVID-19 patients. Overall, the role of the IL-2 cytokine family in COVID-19 is complex and requires further investigation to develop targeted therapies and improve patient outcomes.

## Figures and Tables

**Table 1:** The role of the IL-2 cytokine family in COVID-19.

Sample	Cytokine	Changes in COVID-19 patients	Outcome in disease	Reference
Seventy-four COVID-19 patients with pulmonary fibrosis	TNF-*α*, TGF- *β*1, amphiregulin, IL-2, and EGFR	↑TNF-*α* other cytokines no changes	Association with severity of COVID-19 and pulmonary fibrosis	[58]

Twelve COVID-19 patients with *Aspergillus fumigatus* and *Rhizopus arrhizus* antigens	IFN-*γ*, IL-2, CXCL9, CXCL10	↓IFN-*γ*, IL-2, CXCL9, CXCL10	↓ T-cell and innate immunity function, weakened anti-mold immune responses	[61]

Two patients with a brain tumor and COVID-19	IL-13, IFN-*γ*, and IL-2	↑IL-13, IFN-*γ*, and IL-2	Activation of the microglia and an exacerbated immune response; disruption of BBB	[62]

One hundred three patients with COVID-19	IP-10, IL-4, IL-2	↑IP-10, IL-4, IL-2	Critical COVID-19 outcomes	[63]

One hundred thirty COVID-19 patients and 16 control group	IL-6, IL-15	↑IL-6, IL-15	Extended hospitalization and lymphopenia in COVID-19 patients	[64]

One hundred forty-six COVID-19 patients	Resistin, IL-6, IL-8, IL-15, MCP-1 and TNF-*α*	↑Resistin, IL-6, IL-8, IL-15, MCP-1 and TNF-*α*	Prediction of invasive mechanical ventilation	[66]

Sixty-three COVID-19 patients and 33 control group	IFN-*γ*, IL-5, IL-8, IL-9, IL-17, TGF-*β*	↑IFN-*γ*, TGF-*β*, IL-17, IL-8	TGF-*β* can be a predictive prognosis factor	[59]

Thirty COVID-19 patients and 10 control group	IFN, TNF, IL-21	↑ IFN, TNF, IL-21	Poor prognosis and disease severity	[65]

Fifty-four patients with COVID-19	IL-2, IL-2R	↓IL-2R, ↑IL-2 in severe patients and ↓in critical patients	CD8+ T cell and lymphocyte decrease through JAK1–STAT5 in critical patients	[70]

Six COVID-19 patients and 10 control group	IL-4, IL-13, TGF-*β*	↑IL-4	Lung remodeling	[67]

^*∗*^BBB, blood–brain barrier; EGFR, estimated glomerular filtration rate.

**Table 2:** The role of different interventions in targeting the IL-2 cytokine family in COVID-19.

Intervention	Samples	Targeted cytokine	Cytokine changes	Clinical/experimental outcome	Reference
Nutrition Bio-shield Superfood 2 g/day for 4 weeks	Forty-seven patients with COVID-19 (24 intervention; 23 = control)	IL-2, IL-6, IL-17, IFN*γ*, and TNF*α*	↓IL-2, IL-6, and TNF-*α*	Beneficial anti-inflammatory effect in patients	[77]

600 mg Levagen + twice daily for 4 weeks	Sixty patients with COVID-19 (30 intervention; 30 control)	sP-selectin, IL-1*β*, IL-2	↓sP-selectin, IL-1*β*, IL-2	Anti-inflammatory effects in nonhospitalized patients	[79]

Third/fourth vaccination with Comirnaty® and Spikevax	Thirty-two kidney transplant patients after the booster injection	IFN-*γ*, IL-2	↓IFN-*γ*, ↑IL-2	Partly protection of patients with antibody release and IL-2 production	[80]

Two doses of COVID-19 mRNA-based vaccine (BNT162b2 (Pfizer-BioNTech) or mRNA-1273 (Moderna))	Forty-three MS patients and 34 controls	IFN*γ*, IL-2	↑IFN-*γ*, IL-2	Partial adaptive immune response to COVID-19 vaccination	[81]

Single dose COVID-19 vaccination with inactivated vaccines (CoronaVac, BBIBP-CorV, or WIBP-CorV)	Fifty-one COVID-19 recovered subjects and 63 healthy individuals	IFN*γ*, IL-2, TNF-*α*	↑IFN-*γ*, IL-2, TNF-*α*	Robust humoral and cellular immune response	[82]

Single dose COVID-19 vaccination with BNT162b2	Forty patients with rheumatic diseases and 24 healthy patients	IFN*γ*, IL-2, TNF-*α*	No difference between two groups in IFN*γ*, IL-2, TNF-*α*	No changes in immune response in patients under immunosuppressive treatment	[84]

mACE2-CAR_sIL15 NK cell therapy	A549 cell line and Humanized K18-hACE2 mouse model	IL-15, IFN-*γ*, TNF*α*	↑IL-15, IFN-*γ*, TNF*α*	Decreased viral load and prolonged survival	[88]

First and second doses of BNT162b2 mRNA	Sixty-three COVID-19 patients	IFN-*γ*, IL-15, IP-10/CXCL10	↑IFN-*γ*, IL-15, IP-10/CXCL10	Effective immune response to the vaccine	[83]

3–10 *µ*g/kg IL-7	Twelve COVID-19 patients	TNF-*α*, IL-6, IL-1*β*	No changes	Restored lymphocyte count and disease severity	[89]

^*∗*^MS: multiple sclerosis.

## Data Availability

All data generated or analyzed during this study are included in this published article.
